# Fatty Acid Prediction in Bovine Milk by Attenuated Total Reflection Infrared Spectroscopy after Solvent-Free Lipid Separation

**DOI:** 10.3390/foods10051054

**Published:** 2021-05-11

**Authors:** Christopher Karim Akhgar, Vanessa Nürnberger, Marlene Nadvornik, Margit Velik, Andreas Schwaighofer, Erwin Rosenberg, Bernhard Lendl

**Affiliations:** 1 FG Environmental Analytics, Process Analytics and Sensors, Institute of Chemical Technologies and Analytics, Technische Universität Wien, Getreidemarkt 9, 1060 Vienna, Austria; christopher.akhgar@tuwien.ac.at (C.K.A.); marlene.nadvornik@student.tuwien.ac.at (M.N.); andreas.schwaighofer@tuwien.ac.at (A.S.); 2 Competence Center CHASE GmbH, Altenberger Straße 69, 4040 Linz, Austria; vanessa.nuernberger@chasecenter.at; 3 Agricultural Research and Education Center Raumberg-Gumpenstein, Altirdning 11, 8952 Irdning-Donnersbachtal, Austria; margit.velik@raumberg-gumpenstein.at; 4 FG Instrumental and Imaging Analytical Chemistry, Institute of Chemical Technologies and Analytics, Technische Universität Wien, Getreidemarkt 9, 1060 Vienna, Austria; egon.rosenberg@tuwien.ac.at

**Keywords:** mid-infrared spectroscopy, attenuated total reflection, bovine milk, fatty acids, partial least squares

## Abstract

In the present study, a novel approach for mid-infrared (IR)-based prediction of bovine milk fatty acid composition is introduced. A rapid, solvent-free, two-step centrifugation method was applied in order to obtain representative milk fat fractions. IR spectra of pure milk lipids were recorded with attenuated total reflection Fourier-transform infrared (ATR-FT-IR) spectroscopy. Comparison to the IR transmission spectra of whole milk revealed a higher amount of significant spectral information for fatty acid analysis. Partial least squares (PLS) regression models were calculated to relate the IR spectra to gas chromatography/mass spectrometry (GC/MS) reference values, providing particularly good predictions for fatty acid sum parameters as well as for the following individual fatty acids: C10:0 (R^2^_P_ = 0.99), C12:0 (R^2^_P_ = 0.97), C14:0 (R^2^_P_ = 0.88), C16:0 (R^2^_P_ = 0.81), C18:0 (R^2^_P_ = 0.93), and C18:1cis (R^2^_P_ = 0.95). The IR wavenumber ranges for the individual regression models were optimized and validated by calculation of the PLS selectivity ratio. Based on a set of 45 milk samples, the obtained PLS figures of merit are significantly better than those reported in literature using whole milk transmission spectra and larger datasets. In this context, direct IR measurement of the milk fat fraction inherently eliminates covariation structures between fatty acids and total fat content, which poses a common problem in IR-based milk fat profiling. The combination of solvent-free lipid separation and ATR-FT-IR spectroscopy represents a novel approach for fast fatty acid prediction, with the potential for high-throughput application in routine lab operation.

## 1. Introduction

Milk is among the fastest growing agricultural commodities, with a worldwide production volume of more than 8.5 × 10^6^ tons per annum, and an expected yearly growth rate of 1.6% until 2029 [1]. Bovine milk accounts for approximately 81% of total milk production, and is considered to be one of the most nutritionally complete foods, with a typical gross composition of 3.9% fat, 3.3% protein, and 4.6% lactose [2]. Milk fat predominantly consists of triglycerides, containing more than 400 different fatty acids, but only 15 of them with relative shares of 1% or higher. The largest fraction are saturated fatty acids (SAT, approximately 70%), followed by monounsaturated fatty acids (MONO, approximately 25%), and polyunsaturated fatty acids (PUFA, approximately 5%) [3]. Individual fatty acid content in milk is influenced by different factors, such as animal genetics, stage of lactation, and feed intake [4]. Most controversies regarding the health effects of dairy products are associated with lipid composition [5]. SAT especially are often related with harmful effects such as coronary heart disease, while substitution with PUFA might reduce the risk of such disease [6].

Gas chromatography (GC) is the gold standard for milk fatty acid profiling, offering high accuracy combined with maximum sensitivity [7]. Some of the major drawbacks are, however, the essential derivatization step prior to analysis, high costs, and significant time consumption, thus restricting its use for industrial purposes to a few samples from large batches. The demand for rapid, low-cost, high-throughput fatty acid profiling methods is consequently increasing with growing milk production.

Mid-infrared (IR) spectroscopy is a powerful tool for bioanalytical applications [8], which has been demonstrated to present a rapid, label-free alternative to well-established chromatographic methods for the analysis of dairy products [9]. Specific absorption bands, arising from the rotational–vibrational transitions of molecules, allow for compound identification as well as quantification. Important nutritional parameters such as lactose, total fat, and total protein content are routinely detected using the commercially available MilkoScan (Foss, Hillerød, Denmark), a Fourier-transform infrared (FT-IR) spectrometer specifically developed for the analysis of dairy products [10]. Furthermore, novel laser-based mid-IR transmission spectroscopy shows high potential for the quantification of individual proteins in bovine milk [11,12,13,14].

Attenuated total reflection (ATR) is a prominent alternative probing technique to transmission mode. Here, the incoming IR light is totally reflected in an optically denser ATR element at the interface with a medium of lower optical density. This leads to an evanescent field that can interact with the sample at typical penetration depths of up to 2 µm per reflection [15]. The sample is placed directly on top of the ATR element, allowing for quick and robust measurements of troublesome liquid matrices, such as oils [16,17].

Substantial effort has been put into investigating the potential of mid-IR transmission spectroscopy for milk fatty acid profiling [18,19,20,21,22,23,24]. Here, multivariate chemometric models based on partial least squares (PLS) were established in order to relate mid-IR absorbance spectra acquired from whole milk using the MilkoScan to GC reference data. These studies report good accuracy in predicting the absolute concentrations of certain fatty acids in milk, especially those available in high concentrations, such as C14:0, C16:0, and C18:1. It has been reported that these predictions, however, are most likely based on covariation structures between individual fatty acids and the total fat content, which may change with factors such as breed and feed [25]. When results are stated as relative fatty acid content in milk fat, they appear significantly poorer.

As an alternative approach, dry film FT-IR spectroscopy was introduced [26]. Here, small milk samples were transferred into well plates, dried in a desiccator, and subsequently measured in transmission mode. Multivariate calibrations showed better results than those obtained from direct transmission measurements of whole milk. Here, the lipid preconcentration step was expected to contribute to a major part of the gained prediction improvements. Fine spectral differences associated with fatty acid composition might, however, still be hidden by overlapping absorption bands arising from other major milk components, such as proteins and carbohydrates. Hence, it is of major interest to investigate techniques for lipid separation prior to spectral acquisition, in order to enable improved prediction efficiency and avoid covariation structures with the total fat content.

Classical solvent–solvent extraction methods [27,28] are considered to be reliable for the quantitative separation of lipids from food and animal tissues. Moreover, specific methods for milk fat extraction have been developed, standardized, and are today extensively used in routine lab operation [29,30]. These methods, however, require large amounts of hazardous organic solvents, and are vastly time consuming. A novel, more rapid method using smaller amounts of organic solvents with shorter exposure times allows for the milk fat separation of approximately 20 samples in 30 min [31]. Alternatively, methods based on two centrifugation steps have been successfully applied to obtain pure milk fat without the use of organic solvents [32,33]. Thorough method validation using GC shows that there is no difference in relative fatty acid composition in the obtained lipid fraction compared to standard solvent–solvent extraction. These methods are consequently ideal for applications that require a representative part the of milk lipids instead of quantitative total fat extraction.

The aim of this study is to show the potential of ATR-FT-IR spectroscopy combined with rapid, solvent-free lipid separation for milk fatty acid profiling. The information content of ATR-IR spectra recorded after lipid separation was compared with the IR transmission spectra of whole milk. By performing multivariate PLS analysis, good prediction accuracy could be obtained for individual fatty acids and relevant sum parameters. PLS calibration equations were optimized based on evaluation of the importance of individual wavenumbers to the multivariate models. Cross-correlations between individual fatty acids and total fat content were inherently avoided by the employed approach. The obtained results indicate several clear advantages over conventional FT-IR transmission spectroscopy of whole milk, revealing high potential for future high-throughput applications.

## 2. Materials and Methods

### 2.1. Milk Samples

Forty-five milk samples were collected from the same number of cows in Austria (AREC Raumberg-Gumpenstein, in mid-September 2020), containing two different cattle breeds (¾ Holstein Friesian and ¼ Simmental) and three feeding groups. At the time of sample collection, the averages (±standard deviation) of the milk yield, days in milk, and lactation number were 20.7 ± 5.73 kg per day, 184 ± 5.73 kg days in milk, and 3.6 ± 2.15, respectively, ensuring a variety of milk fat composition. The diets of the three feeding groups were based upon ad libitum allowance of a forage mixture, which consisted of 40% grass silage, 30% maize silage, and 30% hay on a dry matter basis. The pelleted concentrate mixture (0%, 20%, and 40% of total feed intake, respectively) consisted of 25% maize, 24% barley, 8% wheat, 8% molasses, 5% wheat bran, 15% soy meal, and 15% rapeseed meal. A pooled sample from morning and evening milk was collected from each cow. Unhomogenized raw milk samples were immediately stored at −80 °C without further conservation until 1 day before fat separation. A homogenized whole milk sample was purchased from an Austrian retailer and used to acquire a mid-IR transmission reference spectrum of whole milk.

### 2.2. Fat Separation

Milk fat separation was carried out according to the rapid two-step centrifugation method proposed by Feng et al. [32] and modified by Luna et al. [33]. Frozen milk samples were thawed overnight at 4 °C and subsequently tempered at room temperature for at least 20 min. Thirty milliliter aliquots were transferred into falcon tubes and centrifuged at 17,800× *g* for 30 min at 20 °C in a Sigma 3–18k centrifuge (Sigma Laborzentrifugen GmbH, Osterode am Harz, Germany). The fat-cake layer was transferred into microtubes and centrifuged at 19,300× *g* for 20 min at the same temperature, resulting in three separate layers. The upper lipid layer was removed and used for FT-IR and gas chromatography/mass spectrometry (GC/MS) measurements.

### 2.3. GC/MS Analysis

Standard solutions, containing 20 mg of milk fat per mL of dichloromethane, were prepared. For the derivatization of fatty acids, an aliquot of 50 µL of the standard solution was transferred to a pre-cooled 1.5 mL GC vial with a 0.2 mL micro insert. Fifty microliters of internal standard (C17:0) and the same amount of trimethylsulfonium hydroxide (TMSH, 0.25 M in MeOH, Supelco, Vienna, Austria) solution were added, and the vial was capped immediately. Each vial was vortexed for 5 s, and then heated for 15 min at 70 °C to complete derivatization.

A GC instrument (Shimadzu GC-2010) equipped with a ZB-FAME column (30 m, 0.25 mm I.D., 0.20 µm film thickness; Phenomenex, Aschaffenburg, Germany) coupled with a mass spectrometer (GCMS-QP2010 Plus, Shimadzu, Kyoto, Japan) was used to determine fatty acid content and profile. One microliter samples were injected in split mode (split 100:1) using a Shimadzu AOC-5000 Plus autosampler. The injector temperature was 250 °C. The purge flow was set to 3 mL/min and the column flow to 2.14 mL/min. The oven program was 40 °C initially, held for 3 min, then increased by 10 °C/min to 100 °C, and further increased by 2 °C/min to 200 °C. The transfer line temperature of the mass spectrometer was kept at 200 °C, as was the ion source temperature. After a solvent vent of 2.7 min, the detector voltage was set to 1.05 kV, and the samples were measured in scan mode (35–500 *m/z*).

For quantitative analysis, a method was developed and calibrated using a 37-component FAME mix certified reference material (TraceCERT^®^, Supelco, Vienna, Austria). Calibration samples were prepared in the concentration range 20–600 mg/L. Quantitative analysis was based on the evaluation of the quantifier ion peak area for each FAME, provided that the ratio of quantifier and qualifier ions was within acceptable limits. Retention times and qualifier and quantifier ions for each analyte are reported in Table S1 of the Supplementary Materials.

### 2.4. FT–IR Measurements

ATR–FT–IR measurements were performed using a Bruker Tensor 37 FT-IR spectrometer (Ettlingen, Germany) equipped with a mercury cadmium telluride (MCT) detector (D* = 4 × 10^10^ cm Hz^0.5^ W^−1^ at 9.2 μm). The spectrometer was constantly flushed with dry air in order to reduce the influence of water vapor from the atmosphere. One drop of pure milk fat extract was manually placed onto a Platinum ATR single-bounce element (Bruker, Ettlingen, Germany). Measurements were performed with a spectral resolution of 2 cm^−1^, between 600 and 4000 cm^−1^ in double-sided, forward–backward acquisition mode. A Blackman–Harris 3-term apodization function and a zero-filling factor of 2 were used to calculate the final spectra. One hundred and twenty-eight scans were averaged per spectrum, leading to an acquisition time of fifty-two seconds. After each spectral acquisition, the ATR surface was cleaned with isopropanol and dichloromethane consecutively until recovery of the baseline signal. Transmission measurements were performed using the same instrument parameters, by injecting homogenized whole milk into a flow cell equipped with two CaF_2_ windows and a 37 µm-thick spacer. The software package OPUS 7.2 (Bruker, Ettlingen, Germany) was used for evaluation of the spectral data.

### 2.5. Data Analysis

Multivariate data analysis was performed in MATLAB R2020a (Mathworks Inc., Nattick, MA, USA) using PLS Toolbox 8.9 from Eigenvector Research Inc. (Wenatchee, WA, USA). All ATR-IR absorbance spectra were identically preprocessed by calculation of 2nd derivative spectra, using a Savitzky–Golay filter (window = 15 points) and mean centering. The applied wavenumber range was individually selected for each parameter, based on the selectivity ratio (SR) [34]. Preprocessed FT-IR spectra and GC/MS reference values were used to develop partial least squares (PLS) regression models. Model performance was estimated by applying a contiguous blocks cross-validation with 10 data splits, using the full dataset. Furthermore, external validation was applied by randomly dividing the dataset into a calibration set of 30 samples, and an external validation set, containing 15 samples. Characteristic statistical parameters were calculated to evaluate model performance.

## 3. Results and Discussion

### 3.1. Comparison of IR Spectra of Whole Milk and Separated Milk Fat Fraction

Milk fat triglycerides show distinctive mid-IR absorption bands that are influenced by factors such as fatty acid chain length and degree of saturation [35]. Due to this high sensitivity, mid-IR spectroscopy-based prediction of milk fatty acid composition has been reported on multiple accounts and in different implementations. Particularly, the commercially available MilkoScan instrument is widely used for the direct spectral acquisition of whole milk [9]. With this device, FT-IR transmission spectra with a path length of 37 µm are recorded. A limitation of this approach, however, is the limited spectral information in certain wavenumber regions, which can be circumvented by separating the milk fat fraction from the complex milk matrix.

**Figure 1:**
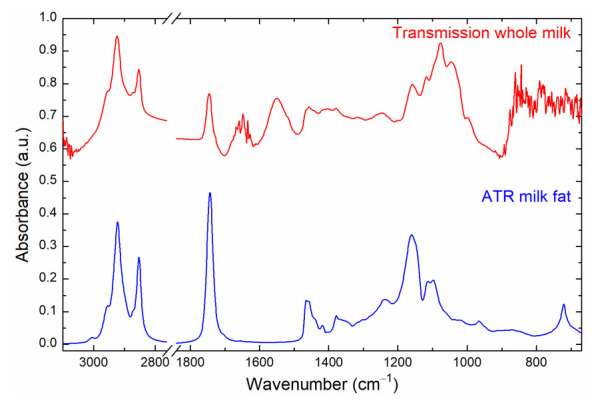
Comparison between the ATR-IR absorbance spectrum of separated milk fat (blue) and whole milk recorded in transmission mode with a CaF_2_ cell with an optical path length of 37 µm (red). The spectral range between 1850 and 2750 cm^−1^ was removed due to lack of relevant information.

In this work, milk fat was separated according to a rapid two-step centrifugation method [33]. It was shown that the hereby obtained lipid fraction possessed a representative fatty acid composition for the whole milk sample [32,33]. In addition to reduced workload and high throughput, the applied separation method excels in that it completely avoids the use of potentially hazardous or toxic solvents. This characteristic is particularly beneficial for subsequent mid-IR spectroscopy, as small residues of organic solvents can already lead to distinctive absorption bands that hide spectral details of the sample. This is specifically relevant in the present application, because the routinely employed solvents for milk fat extraction often exhibit the same functional groups (e.g., CH_2_, CH_3_) and, consequently, IR bands as lipids. Reproducible spectral acquisition after the immediate drying of solvent-based extracts on straight surfaces, such as the ATR crystal, is moreover restricted by the coffee-ring effect [36], which requires complex instrumentation to be avoided [37]. The combination of lipid separation and ATR-FT-IR spectroscopy provides the advantage that very small amounts of sample can be measured in a robust environment by placing them directly onto the active element. In the present study, one drop of the milk fat fraction was sufficient to cover the surface of the ATR crystal and to record representative absorbance spectra. Characteristic mid-IR bands of milk fat are listed in Table 1. Figure 1 displays the typical absorbance spectra of separated milk fat measured in ATR mode (blue) and whole milk recorded in transmission mode with a CaF_2_ cell and an optical path length of 37 µm (red). Here, the wavenumber range between 1850 and 2750 cm^−1^ was removed due to lack of information in this region. Visual inspection reveals that the information content of the two IR spectra is significantly different. The whole milk sample also contains vibrational bands from other major components of milk, such as lactose and other carbohydrates (approximately 1000–1480 cm^−1^) and proteins (amide II band: 1500–1600 cm^−1^) [38].

The high absorbance of these components adversely affects the evaluation of the significantly lower absorbances originating from fatty acids in these spectral regions. In this context, the low wavenumber region overlapping with carbohydrate absorption bands in particular has proven to be important for the quantitative prediction of individual fatty acids (see Section 3.2.2). Furthermore, for transmission measurements with milk, large transmission paths (>30 µm) are required in order to prevent clogging of the cell because of the high viscosity and complex matrix of milk [40]. However, at these high optical paths, water (HOH bending band: 1643 cm^−1^) totally absorbs the irradiated IR light, and thus the spectral region between 1600 and 1700 cm^−1^ is not accessible. Moreover, CaF_2_, the typical window material used for transmission measurements of bovine milk, has its absorption edge at approximately 1000 cm^−1^ [41], meaning that IR bands at lower wavenumbers are not accessible using this approach. In this inaccessible spectral region for transmission measurements, there are located the C–H out-of-plane band at 966 cm^−1^ and the C–H rocking band at 722 cm^−1^, which are well resolved in ATR-IR spectra of lipids. Due to these limitations, for the purpose of the determination of fatty acids, the related spectral features are better resolved in the ATR spectra, thus highlighting the advantage of the lipid separation step.

### 3.2. Predicting Fatty Acid Content by Mid-IR Spectroscopy

#### 3.2.1. Partial Least Squares Analysis

**Figure 2:**
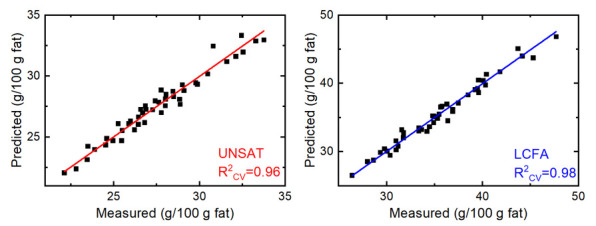
Relationship between measured (GC/MS) and predicted (cross-validation, FT–IR) fatty acid content in g/100 g fat for unsaturated fatty acids (UNSAT, **left**) and long-chain fatty acids (LCFA, **right**).

Individual PLS1 models were calculated to predict the most abundant fatty acids as well as the relevant sum parameters. PLS is a multivariate statistical approach, capable of calculating linear regression models from highly correlated variables, such as those usually found in spectroscopic data [42]. In the present study, the relationship between the recorded ATR absorbance spectra (x-matrix) and the GC/MS reference fatty acid concentration (y-matrix) was calculated. A preprocessing routine, combining second derivative spectra with mean centering, was applied in order to achieve optimal results. Moreover, the included wavenumbers were individually selected for each model (see next chapter). Table 2 provides an overview of the obtained statistical parameters. The root mean square error of calibration (RMSEC) and the calibration coefficient of determination (R^2^) were calculated by using the full dataset of available milk samples (*n* = 45) in order to assess the quality of the calibration equations. For the visualization of the calibration equations, Figure 2 shows the relationship between the measured and predicted concentrations on the examples of unsaturated fatty acids (UNSAT, red) and long-chain fatty acids (LCFA, blue). In the optimal case, all points would lie on the regression line, while those above and below indicate over- and underestimation of ATR-based predictions compared to GC/MS reference values. The small deviation of individual data points from the regression line, as well as the obtained R^2^-values of 0.99, indicate highly linear relationships and very good description of the data by the model. Evaluation of prediction efficiency was performed using contiguous blocks cross-validation with 10 data splits, revealing the root mean square error of cross-validation (RMSECV) and the cross-validation coefficient of determination (R^2^_CV_). Furthermore, external validation was applied by randomly dividing the dataset into a calibration set (*n* = 30) and an external validation set (*n* = 15). Table S2 shows the obtained statistical parameters for the reduced calibration set of 30 samples. The achieved root mean square error of prediction (RMSEP) and prediction coefficient of determination (R^2^_p_) from the external validation (Table 2) show similar results to the cross-validation, using the whole dataset, indicating high robustness of the calculated prediction models. The optimal number of latent variables (LVs), based on the lowest RMSECV, was between three and eight, which is reasonable for milk fat, which contains a high number of different fatty acids that can cause spectral variability in the system under study. Good prediction efficiencies were obtained for the important health related parameters SAT (R^2^_CV_ = 0.94, R^2^_P_ = 0.95) and UNSAT (R^2^_CV_ = 0.96, R^2^_P_ = 0.95). Further subclassification showed good prediction for MONO (R^2^_CV_ = 0.95, R^2^_P_ = 0.94), while the much lower concentrated PUFA were predicted with moderate accuracy (R^2^_CV_ = 0.61, R^2^_P_ = 0.27). Moreover, sum parameters regarding fatty acid chain length were calculated. Highly concentrated medium-chain fatty acids (MCFA, C12-C16) and LCFA (C17 and higher) were predicted with excellent accuracy (R^2^_CV_ = 0.95/0.98, R^2^_P_ = 0.97/0.99), whereas the lower concentrated group of short-chain fatty acids (SCFA, C4-C10) was predicted with moderate accuracy (R^2^_CV_ = 0.64, R^2^_P_ = 0.83). In the case of individual fatty acid content, excellent predictions (R^2^_CV_ > 0.92, R^2^_P_ > 0.93) were achieved for C10:0, C12:0, C18:0, and C18:1cis, while feasible predictions (R^2^_CV_ > 0.84, R^2^_P_ > 0.81) were obtained for C14:0 and C16:0.

An important parameter that can influence the quality of calibration equations is the number of applied samples. Previously, it has been shown that that use of many different samples can increase the predictability of milk fat composition [20]. For the present study, only a limited set of 45 samples was available. Nevertheless, the achieved results are clearly better than those reported for MilkoScan measurements [18,20] and ATR-FT-IR measurements of whole milk without fat separation [43], when final concentrations are stated in g/100 g fat. Moreover, the presented results are comparable to those obtained from the dry film approach, where a much higher number of samples (*n* = 219) was used [26]. Supposedly, an explanation for the more robust fatty acid prediction enabled by the presented approach is the high accessibility to significant spectral features of the fat fraction compared to (dried) whole milk samples when using ATR-FT-IR spectroscopy, as discussed in the previous section. We expect that even better results can be achieved with the herein presented approach of lipid separation followed by ATR-FT-IR spectroscopy when a larger number of different milk samples are available, indicating high potential for future applications. Finally, it should be noted that additional fatty acids were quantified using the GC/MS reference method, which were present at low concentrations (<2 g/100 g milk fat). However, due to the limited sensitivity of IR spectroscopy and the small sample set, it was not possible to obtain reliable prediction equations for these analytes.

#### 3.2.2. Selection of Wavenumber Range Based on Selectivity Ratio

FT–IR spectra were recorded in the wavenumber range between 600 and 4000 cm^−1^ in order to acquire the maximal amount of information within the mid-IR region. However, for each PLS1 model, the applied spectral region was individually selected, based on the selectivity ratio (SR). The SR is a visualization tool to identify important variables in a multivariate data set for predicting the target variable. A detailed description and mathematical definition can be found elsewhere [34,44]. Briefly, it can be defined as the ratio between explained and unexplained variance for each variable of the model. In the case of mid-IR spectroscopy, the SR is useful for determining specific spectral features with high correlation to the parameter of interest [45].

**Figure 3:**
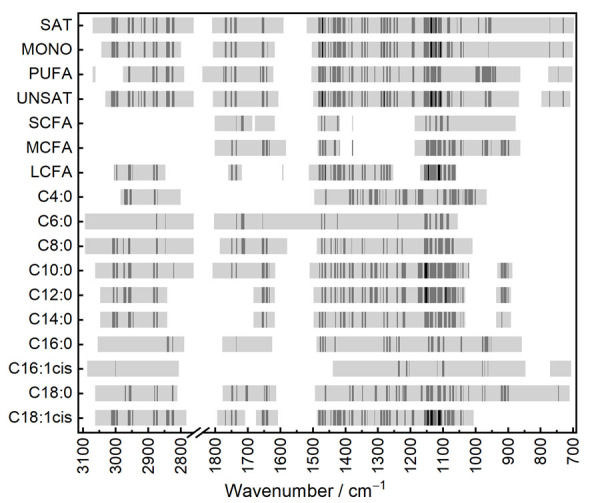
Heatmap, showing spectral regions included for each PLS model in greyscale. Bright grey: selectivity ratio (SR) = 0–0.5; dark grey: SR = 0.5–5; black: SR = 5–15. The spectral range between 1850 and 2750 cm^−1^ was removed due to lack of relevant information. SAT: saturated fatty acids; MONO: monounsaturated fatty acids; PUFA: polyunsaturated fatty acids; UNSAT: unsaturated fatty acids; (C4–C10); MCFA: medium-chain fatty acids (C12–C16); LCFA: long-chain fatty acids (C17 and higher).

In this work, the following wavenumber regions without relevant information regarding fatty acid composition were removed for all PLS models: 600–700, 1800–2750, and 3100–4000 cm^−1^. The remaining wavenumbers were individually selected for each target parameter. Figure 3 displays the included wavenumbers for each calibration model as calculated from the full dataset. Here, brighter regions indicate wavenumbers with low SR, whereas dark areas highlight those with high SR. The figure shows that the significant wavenumbers are highly different between sum parameters that describe fatty acid saturation degree and those that describe chain length. The wavenumber range close to 3005 cm^−1^ has a high SR for SAT, MONO, and UNSAT, while this region is not important for predicting the chain length. This result seems reasonable, because the associated absorption band arises from the C–H stretching vibration of the cis double bond. Moreover, the spectral region between approximately 2700 and 3000 cm^−1^, covering several C–H stretching absorption bands, plays an important role in predicting the saturation degree. This relationship was also observed by Christy et al. [46], who predicted the saturation degree in different edible oils using ATR-FT-IR spectroscopy.

The spectral range near 1643 cm^−1^, not accessible in transmission measurements of whole milk, also contains relevant information regarding the saturation degree. Even though the C=C stretching vibration of unsaturated carbonyl compounds is barely IR-active [47], a weak related absorption band at approximately 1655 cm^−1^ can be observed in the ATR–IR spectra of some lipids. High SR in terms of saturation degree was also obtained in the spectral region between approximately 1050 and 1500 cm^−1^, which distinctly overlaps with other major components in whole milk transmission spectra. Moreover, the C–H out-of-plane band at 966 cm^−1^ and the C–H rocking band at 722 cm^−1^, inaccessible in transmission measurements using CaF_2_ windows, contain information regarding the saturation degree. PLS models predicting sum parameters concerning chain length showed particularly good results for MCFA and LCFA. Here, the spectral region below 1500 cm^−1^ especially contains several wavenumbers with high SR, indicating important information. The effect of fatty acid chain length on this spectral region was thoroughly investigated by Jones [48], concluding that various small band shifts appear with changing chain length. For predicting individual fatty acids, similar spectral features are important.

Generally, fatty acids with high prediction accuracy, such as C10:0 and C18:1cis, show distinct wavenumber regions with high SR, while fatty acids with weaker prediction, such as C6:0 and C16:1cis, show lower SR.

In conclusion, all spectral regions with medium and high SR can be assigned to absorption bands specific to fatty acids. This verification step is crucial in order to confirm that the information content of calibration equations is based on real absorbance of fatty acids rather than on incidental correlations. While important spectral features are well resolved in the ATR spectra of separated milk fat, a great part of them is hardly accessible or completely inaccessible in whole milk transmission spectra. This evaluation, involving the identification of relevant wavenumbers, thus demonstrates the benefit of using ATR-FT-IR spectroscopy on the milk fat fraction.

#### 3.2.3. Evaluation of Covariation Structures

Due to the performed analysis of the milk fat fraction after separation from the milk matrix, the herein presented approach enables us to state the obtained results in terms of g fatty acid/100 g fat. When comparing the results to other works reporting on mid-IR-based predictions of fatty acids in bovine milk, it should be noted that most authors stated their concentrations in g/100 mL of whole milk. However, it has been demonstrated that this good prediction accuracy is indirect, and primarily based on covariation between individual fatty acids and total fat content, whose dependencies may change with factors such as breed and feed [25]. Covariance is a measure of the degree of association between two random variables [49]. This issue was outlined by showing that PLS models calculated from the milk spectra of a specific cattle breed result in biased predictions when applied to another breed, due to different covariation structures. To further highlight this issue, prediction was performed using a calibration set compiled from skimmed milk spiked with three of the most abundant fatty acids at concentration values avoiding cross-correlations. This approach resulted in significantly poorer models than those obtained for unspiked whole milk. Prediction models, calculated from the same samples, stating the relative fatty acid concentrations in g/100 g fat are, consequently, significantly weaker.

**Figure 4:**
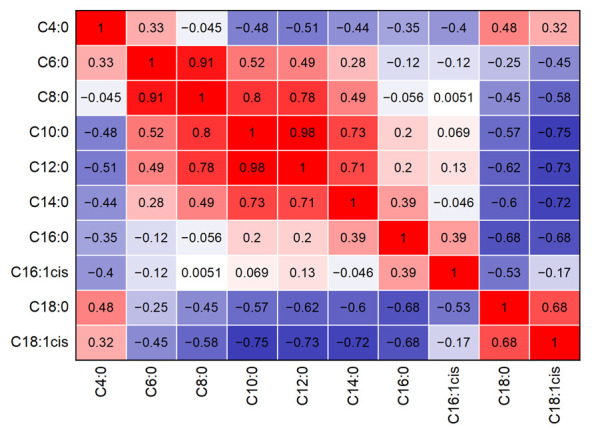
Cross-correlation matrix of pairwise correlations between individual fatty acids from GC/MS reference measurements. Red color indicates positive correlation, whereas blue color indicates negative correlation.

The presented approach, based on ATR-IR measurements, does not have the purpose of providing information regarding total milk fat content, but rather of investigating the relative fatty acid profile. In this way, possible covariation structures to total fat content are inherently eliminated. Consequently, meaningful comparison to published results is only useful for reports where the predicted fatty acid concentrations are also stated relative to milk fat content. Correlations between individual fatty acids are, however, still a great challenge in the spectroscopic prediction of milk fat composition. For this reason, Figure 4 shows a cross-correlation plot of the relative fatty acid content based on the GC results of the applied dataset. For this purpose, Pearson correlation coefficients indicate highly positive correlation (+1), no correlation (0) and highly negative correlation (-1). Here, positively correlated fatty acids are marked in red, whereas those of negatively correlated fatty acids are marked in blue. The plot reveals that most pairwise correlations are small, thus indicating that the predicted concentrations of these fatty acids resulted more from corresponding IR information than from correlations to other fatty acids. Short-chain SAT with similar chain length (i.e., C6:0 and C8:0, C8:0 and C10:0) are, however, highly correlated. These correlations can also be seen in their similar SR profiles in Figure 3. Consequently, grouping similar fatty acids into sum parameters (i.e., SCFA), as was done in this study, is highly beneficial in order to avoid high cross-correlations. Afseth et al. [26] observed comparable correlations between fatty acids in milk with similar chain length, and highlighted that these internal correlations can be used for reliable predictions as long as they are within some degree of certainty valid for future samples.

## 4. Conclusions

In this work, a new mid-IR-based approach for predicting the fatty acid composition of bovine milk was introduced. A rapid, solvent free, two-step centrifugation method was employed in order to obtain representative milk fat fractions. Absorbance spectra of pure lipids were recorded using ATR-FT-IR spectroscopy, and compared to the transmission spectra of whole milk. Fatty-acid-related spectral features were distinctively better resolved in ATR spectra, highlighting the advantage of the preceding lipid separation step. PLS-based multivariate calibration models were calculated in order to relate IR absorbance spectra to relative concentrations of the most abundant fatty acids and sum parameters, obtained via a GC/MS reference method. Prediction efficiency was evaluated by performing cross-validation on the full dataset, as well as by splitting the dataset into a calibration and a validation set. Both methods showed excellent results, indicating high robustness of the models. Particularly high prediction accuracies were obtained for SAT, MONO, UNSAT, MCFA, LCFA, C10:0, C12:0, C14:0, C16:0, C18:0, and C18:1cis. Based on a set of 45 milk samples, the obtained results were clearly better than those reported in literature for whole milk transmission spectra when concentrations were stated in g/100 g fat. The information content of the calibration equations was evaluated by identifying the most important spectral features for predicting individual target variables. Here, relevant wavenumbers were identified based on SR, and successfully assigned to absorbance bands from milk fat. Covariation structures between total fat content and predicted parameters, a common problem in IR-based milk fat profiling, were inherently eliminated with the applied approach. Consequently, the presented method bears several clear advantages over FT-IR transmission spectroscopy of whole milk, revealing its high potential for high-throughput applications. ATR-FT-IR measurements of pure milk fat, including cleaning procedures, can be performed in less than 2 min. When a high number of samples must be analyzed, the two centrifugation steps (30/20 min) can be performed in parallel, and the work flow optimized to provide maximum sample throughput. In the future, the calibration equations might be further improved by using a higher number of different milk samples, whereas additional automatization of the fat separation procedure could facilitate high-throughput operation.

## Figures and Tables

**Table 1:** Characteristic mid-IR absorption bands of milk fat [39].

Wavenumber/cm^−1^	Detectable in Whole Milk *	Group	Mode of Vibration	Functional Group
3005	no	C–H	sym. stretch	-C=CH- (cis)
2953	yes	C–H	asym. stretch	-CH_3_ (aliphatic)
2922	yes	C–H	asym. stretch	-CH_2_- (aliphatic)
2853	yes	C–H	sym. stretch	-CH_2_- (aliphatic)
1743	yes	C=O	stretch	C=O ester
1655	no	C=C	stretch	C=C (unsaturated)
1462	overlapping	C–H	scissoring	-CH_2_- (aliphatic)
1377	overlapping	C–H	sym. deformation	-CH_3_ (aliphatic)
1238	overlapping	C–H	out-of-plane bend	-CH_2_- (aliphatic)
1162	overlapping	C–O	stretch	C-O ester
966	no	C–H	out-of-plane bend	-C=CH- (trans)
722	no	C–H	rocking	-CH_2_- (aliphatic)

* Detection of the absorption band in a whole milk spectrum acquired in transmission mode, using CaF_2_ windows and an optical path length of 37 µm. Abbreviations: sym.: symmetric; asym.: asymmetric.

**Table 2:** Statistical parameters for each individual calibration equation estimating relative individual fatty acid concentration and relevant sum parameters in g/100 g of fat.

		g/100 g Fat						
			Full Dataset (*n* = 45)				Split Dataset (*n*= 30/15)	
Fatty Acid	LVs	Range	RMSEC	RMSECV	R^2^	R^2^_CV_	RMSEP	R^2^_P_
SAT	8	61.6–74.5	0.27	0.66	0.99	0.94	0.8	0.95
MONO	8	19.8–30.3	0.28	0.57	0.99	0.95	0.74	0.94
PUFA	3	2.2–4.2	0.20	0.24	0.73	0.61	0.28	0.27
UNSAT	8	22.1–33.8	0.28	0.58	0.99	0.96	0.74	0.95
SCFA	7	14.2–21.0	0.45	0.78	0.87	0.64	0.67	0.83
MCFA	7	38.1–56.0	0.57	0.96	0.98	0.95	0.85	0.97
LCFA	7	26.4–47.7	0.43	0.76	0.99	0.98	0.65	0.99
C4:0	6	5.4–8.8	0.27	0.42	0.87	0.72	0.49	0.62
C6:0	5	3.1–5.4	0.20	0.31	0.72	0.38	0.24	0.71
C8:0	5	1.5–3.2	0.12	0.16	0.81	0.64	0.11	0.88
C10:0	7	2.1–4.9	0.05	0.11	0.99	0.97	0.10	0.99
C12:0	5	2.0–5.6	0.09	0.16	0.99	0.96	0.19	0.97
C14:0	7	7.4–13.3	0.20	0.49	0.97	0.85	0.48	0.88
C16:0	8	21.1–35.1	0.40	1.05	0.98	0.85	1.4	0.81
C16:1cis	4	1.2–3.9	0.28	0.41	0.73	0.44	0.44	0.39
C18:0	5	5.6–14.6	0.38	0.57	0.97	0.93	0.63	0.93
C18:1cis	8	14.9–27.2	0.22	0.74	0.99	0.92	0.77	0.95

Abbreviations: LVs: latent variables; RMSEC: root mean square error of calibration; RMSECV: root mean square error of cross-validation; RMSEP: root mean square error of prediction; R^2^: calibration coefficient of determination; R^2^_CV_: cross-validation coefficient of determination; R^2^_P_: prediction coefficient of determination; SAT: saturated fatty acids; MONO: monounsaturated fatty acids; PUFA: polyunsaturated fatty acids; UNSAT: unsaturated fatty acids; SCFA: short-chain fatty acids (C4–C10); MCFA: medium-chain fatty acids (C12–C16); LCFA: long-chain fatty acids (C17 and higher).

## Data Availability

The data presented in this study are available on request from the corresponding author.

## Supplementary Materials

The following are available online at https://www.mdpi.com/article/10.3390/foods10051054/s1: Table S1: Retention time, quantifier and qualifier ions for each analyte of the applied GC/MS method; Table S2: Statistical parameters for each individual calibration equation, using a calibration set of 30 samples and a validation set of 15 samples.
