# Supplementary material for: Fatty Acid Prediction in Bovine Milk by Attenuated Total Reflection Infrared Spectroscopy after Solvent-Free Lipid Separation
Source: Foods. 2021 May 11;10(5):1054. doi: 10.3390/foods10051054 (PMC8151219; doi:10.3390/foods10051054)
Supplement: Supplementary file 1 [file foods-10-01054-s001.zip › foods-1183806-supplementary.pdf]

Supplementary material

Fatty Acid Prediction in Bovine Milk by Attenuated Total Reflection Infrared Spectroscopy after Solvent-free Lipid Separation

Christopher K. Akhgar [1], Vanessa Nürnberger [2], Marlene Nadvornik [1], Margit Velik [3], Andreas Schwaighofer [1], Erwin Rosenberg [4] and Bernhard Lendl [1,*]

[1] FG Environmental Analytics, Process Analytics and Sensors, Institute of Chemical Technologies and Analytics, Technische Universität Wien, Getreidemarkt 9, 1060 Vienna, Austria;
christopher.akhgar@tuwien.ac.at (C.K.); marlene.nadvornik@student.tuwien.ac.at (M.N.); andreas.schwaighofer@tuwien.ac.at (A.S.)

[2] Competence Center CHASE GmbH, Altenberger Straße 69, 4040 Linz, Austria; vanessa.nuernberger@chasecenter.at

[3] HBLFA Raumberg-Gumpenstein, Altirdning 11, 8952 Irdning-Donnersbachtal, Austria; margit.velik@raumberg-gumpenstein.at

[4] FG Instrumental and Imaging Analytical Chemistry, Institute of Chemical Technologies and Analytics, Technische Universität Wien, Getreidemarkt 9, 1060 Vienna, Austria; egon.rosenberg@tuwien.ac.at

* Correspondence: bernhard.lendl@tuwien.ac.at; Tel.: +43/1/58801-15140

Table S1. Retention time, quantifier and qualifier ions for each analyte of the applied GC/MS method.

Compound	Retention Time (min)	Quantifier ion (m/z)	Qualifier ion 1 (m/z)	Qualifier ion 2 (m/z)
C4:0	2.795	43	74	71
C6:0	5.729	74	43	87
C8:0	8.519	74	87	43
C10:0	11.3	74	87	43
C11:0	13.185	74	87	43
C12:0	15.457	74	87	43
C13:0	18.164	74	87	43
C14:0	21.2	74	87	43
C14:1	22.833	55	41	74
C15:0	24.482	74	87	43
C15:1 cis	26.148	55	41	74
C16:0	27.902	74	87	43
C16:1 cis	29.092	55	69	41
C17:0	31.332	74	87	43
C17:1 cis	32.485	55	69	41
C18:0	34.789	74	87	43
C18:1 trans	35.332	55	69	41
C18:1 cis	35.594	55	69	41
C18:2 trans	36.922	67	81	95
C18:2 cis	37.605	67	81	55
C18:3 n6	38.993	79	67	80
C1C18:3 n3	40.168	79	67	95
C20:0	41.483	74	87	43
C20:1 cis	42.124	55	69	83
C20:2 cis	43.995	67	81	55
C21:0	44.68	74	87	43
C20:3 n6	45.192	79	67	80
C20:4 n6	46.081	79	91	80
C20:3 n3	46.371	79	67	95
C22:0	47.851	74	87	43
C22:1 cis	48.38	55	69	83
C20:5 (EPA)	48.63	79	91	67
C22:2	50.079	67	81	55
C23:0	50.86	74	87	43
C24:0	53.873	74	87	43
C24:1 cis	54.312	55	69	83
C22:6 (DHA)	55.417	79	91	67

Table S2. Statistical parameters for each individual calibration equation, using set of 30 samples as employed for external validation.

		g/100g fat		
**Fatty acid**	**LVs**	**Range**	**RMSEC**	**R^2**
SAT	8	61.6 - 74.5	0.19	0.99
MONO	8	19.8 - 30.3	0.16	1.0
PUFA	3	2.2 - 4.2	0.17	0.81
UNSAT	8	22.1 - 33.8	0.17	1.0
SCFA	7	14.2 - 21.0	0.30	0.94
MCFA	7	38.1 - 56.0	0.44	0.99
LCFA	7	26.4 - 47.7	0.32	0.99
C4:0	6	5.4 - 8.8	0.19	0.93
C6:0	5	3.1 - 5.4	0.2	0.72
C8:0	5	1.5 - 3.2	0.11	0.79
C10:0	7	2.1 - 4.9	0.04	1.0
C12:0	5	2.0 - 5.6	0.08	0.98
C14:0	7	7.4 - 13.3	0.15	0.98
C16:0	8	21.1 - 35.1	0.22	0.99
C16:1cis	4	1.2 - 3.9	0.3	0.74
C18:0	5	5.6 - 14.6	0.36	0.97
C18:1cis	8	14.9 - 27.2	0.15	1.0

Abbreviations: LVs: latent variables; RMSEC: root mean square error of calibration; R^2: calibration coefficient of determination. SAT: saturated fatty acids; MONO: monounsaturated fatty acids; PUFA: polyunsaturated fatty acids; UNSAT: unsaturated fatty acids; SCFA: short-chain fatty acids (C4-C10); MCFA: medium-chain fatty acids (C12-C16); LCFA: long-chain fatty acids (C17 and higher).
